# Hybrid Nanoparticles Based on Mesoporous Silica and Functionalized Biopolymers as Drug Carriers for Chemotherapeutic Agents

**DOI:** 10.3390/ma17153877

**Published:** 2024-08-05

**Authors:** Federica Curcio, Michela Sanguedolce, Luigino Filice, Flaviano Testa, Gerardo Catapano, Francesca Giordano, Sonia Trombino, Roberta Cassano

**Affiliations:** 1 Department of Pharmacy, Health and Nutritional Sciences, University of Calabria, Via P. Bucci, Arcavacata di Rende, 87036 Cosenza, Italy; federica.curcio@unical.it (F.C.); francesca.giordano@unical.it (F.G.); 2 Department of Mechanical, Energy and Management Engineering, University of Calabria, Via P. Bucci, 87036 Rende, Italy; michela.sanguedolce@unical.it (M.S.); luigino.filice@unical.it (L.F.); gerardo.catapano@unical.it (G.C.); 3 Department of Computer Engineering, Modeling, Electronics and Systems Engineering, University of Calabria, Via P. Bucci, 87036 Rende, Italy; flaviano.testa@unical.it

**Keywords:** mesoporous silica nanoparticles, 5-fluoruracil, silymarin, hyaluronic acid–folic acid, carboxymethyl chitosan–dopamine

## Abstract

Mesoporous silica nanoparticles (MSNs) are promising drug carriers for cancer therapy. Their functionalization with ligands for specific tissue/cell targeting and stimuli-responsive cap materials for sealing drugs within the pores of MSNs is extensively studied for biomedical and pharmaceutical applications. The objective of the present work was to establish MSNs as ideal nanocarriers of anticancer drugs such as 5-FU and silymarin by exploiting characteristics such as their large surface area, pore size, and biocompatibility. Furthermore, coating with various biopolymeric materials such as carboxymethyl chitosan–dopamine and hyaluronic acid–folic acid on their surface would allow them to play the role of ligands in the process of active targeting to tumor cells in which there is an overexpression of specific receptors for them. From the results obtained, it emerged, in fact, that these hybrid nanoparticles not only inhibit the growth of glioblastoma and breast cancer cells, but also act as pH-responsive release systems potentially useful as release vectors in tumor environments.

## 1. Introduction

Polymeric materials and/or ceramics have been extensively studied as materials for use in engineering applications either as substitutes for artificial bone grafts or as controlled release materials. However, when bioactive calcium phosphates are used as transport systems for biologically active molecules, they undergo the so-called burst release phenomenon, which is contraindicated in the treatment of specific diseases, such as cancer [1]. To bypass this problem, attention has been focused on oxide and mesoporous silica systems and specifically on low-temperature processed silica sol–gel materials [2]. Some of the important advantages of these delivery systems are their excellent biocompatibility and ability to control release kinetics, especially in relation to their high surface area and adjustable pore size (2–10 nm) [3,4]. Of all available nanomaterials, porous silica nanoparticles (MSNs) were found to be the best due to their unique properties, including providing great potential for drug uptake and loading within pore channels, an excellent mesoporous structure and adjustable pore size allowing for better control of drug loading and release kinetics, and an easily modifiable surface for controlled and targeted drug delivery to improve therapeutic drug efficacy and reduce toxicity [5,6]. The abundant silanol groups on the MSN surface can be actively functionalized to increase positive or negative charges, correcting hydrophobicity or hydrophilicity, improving targeting functions, or supporting controlled drug release [7]. These modifications can be achieved by the co-condensation of organosiloxane or siloxane, post-synthesis grafting, and molecular imprinting [8]. Recently, functionalized MSNs with organic or inorganic portions have also been described that regulate the release of host molecules triggered by a variety of external stimuli [9], such as chemical stimuli [10], temperature [11], pH changes [12], redox reactions [13], or photoirradiation [14]. This would allow MSNs to be efficiently internalized into cells, without inducing toxicity, affecting cell viability, growth, or differentiation, and escaping from endolysosomal vesicles with ease [15]. The aim of the present work was to create mesoporous silica nanoparticles loaded with drugs with antitumor activity such as 5-fluorouracil and silymarin and coated with functional polymers such as carboxymethyl chitosan and hyaluronic acid functionalized with dopamine and folic acid. The severe systemic toxic effects associated with the use of these anticancer agents have made it imperative to develop targeted systems that reduce systemic cytotoxicity, providing an effective and safe therapy for the treatment of cancer through the administration of a reduced and controlled dose over time. The decision to use carboxymethyl chitosan functionalized with dopamine lies in the properties of the polymer, which is highly biodegradable and biocompatible with organic tissues, and above all is ph-responsive. In fact, in acid ph environments, typical of the tumor environment, the polymer tends to swell easily and favors the release of the biologically active substance. Moreover, dopamine binding would ensure that it actively targets the tumor cells, in which there is an overexpression of specific receptors for it [16] The same mechanism would be activated with regard to hyaluronic acid and folic acid, both of which are able to interact with specific surface markers overexpressed in tumor cells of different forms [17]. The nanoparticles were characterized through several analytical techniques, including low-angle X-ray diffraction (XRD), FTIR spectroscopy, differential scanning calorimetry (DSC), and Dynamic Light Scattering (DLS). Subsequently, MSNs were subjected to release studies under conditions simulating various environments, including tumor environments.

## 2. Materials and Methods

### 2.1. Materials

Cetyltrimethylammonium bromide (CTAB) ≥ 98%, tetraethyl orthosilicate (TEOS) > 97.5%, mesitylene 98%, dimethyl sulfoxide (DMSO) ≥ 99.9%, NaOH, carboxymethylchitosan ((CS), medium molecular weight, deacetylation degree 75–85%, viscosity 200–800 mPa·s) dopamine hydrochloride (DA), and PBS were purchased from Sigma-Aldrich (Milan, Italy). Hyaluronic acid (HA) MW, 300 kDa, was purchased from FarmaLabor, Lithium chloride (LiCl), folic acid (FA), N,N′-Dicyclohexylcarbodiimide (DCC) 95%, and 4-Dimethylaminopyridine (DMAP) 96% were purchased from Carlo Erba Reagents (Milan, Italy).

### 2.2. Instruments

FT-IR spectra were obtained using a Jasco 4200 spectrophotometer Jasco, Milano, Italy). UV-Vis spectra were obtained using a Jasco V-530 UV/Vis spectrophotometer (Jasco, Milan, Italy). Size analyses of the nanoparticles were carried out using a Brookhaven 90 Plus Particle Size Analyzer with light scattering (Holtsville, NY, USA) while scanning electron microscopy (SEM) was carried out using an SEM ZEISS Crossbeam 350. The differential scanning calorimetry (DSC) was performed with DSC 200 PC NETZSCH (NETZSCH, Waldkraiburg, Germany). The samples were freeze-dried by Micro Freeze-drying, Modulyo, Edwards. X-ray diffraction analysis was performed using a Miniflex600 (Rigaku, Tokyo, Japan). The diffraction patterns were collected in the range of 2θ = 1.5–7.0°.

### 2.3. Preparation of MSNs

MSNs were prepared according to the reported procedures in the presence of mesitylene [18]. A total of 2.0 g of CTAB and 14 mL of mesitylene were added to a solution containing 1 L of water and 7 mL of NaOH. After vigorous stirring at 90 °C for 5 h, 10 mL of TEOS was added rapidly into the solution. The reaction was vigorously stirred all night. The white precipitate was separated by vacuum filtration, washed with ethanol several times and then dried overnight under a vacuum at 45 °C to form MSNs as a white powder. As-synthesized materials are usually heated at a rate of 1–2 °C/min to 550 °C for 4–6 h for complete removal of organic templates (CTAB).

### 2.4. Synthesis and Coating of MSNs with Active Substances

For the synthesis of 5-fluoruracil-based MSNs (5-FU/MSNs), and silymarin-based MSNs (SI/MSN), 5-fluoruracil and silymarin (30 mg) was dissolved in DMSO (3 mL). Then calcinated, different aliquots of MSNs (30 mg) were dispersed in each prepared solution and they were left to stir at room temperature in the dark for 24 h to prepare MSNs loaded with the drugs. The polymeric solution based on hyaluronic acid esterified with folic acid (HA-FA) and carboxymethyl chitosan esterified with dopamine (CMCH-DA) solubilized in 1.5 mL di DMSO, was added subsequently and it was stirred at 37 °C in the dark for 6 h. The polymeric substrates, used for the coating, were made according to the procedure reported by Cassano et al. and Serini et al. [19,20,21]. Any precipitate was centrifuged and it was washed with deionized water repeatedly. At the end, the coated and uncoated final products were dried by lyophilization to obtain the products reported in Table 1.

### 2.5. Characterization of Nanoparticles

Various techniques were used to characterize the prepared MSNs. In particular, MSNs were analyzed using SEM, FT-IR, and XRD analysis. SEM was used to determine the shape and surface morphology of the MSNs in a dried state. The nanoparticles were vacuum-coated with graphite prior to analysis. while the water-swollen nanoparticles were analyzed through DLS measurement. XRD patterns were collected using a Miniflex600 powder diffraction system. The presence of amorphous silica was analyzed by the low-angle XRD pattern in the range 2θ = 1.5–7°. The composition of the nanoparticles in each phase was determined by FT-IR spectra.

### 2.6. Drug Entrapping Profile

In order to determine the efficiency and drug loading capacity, the supernatant collected from each centrifuge was analyzed for the different types of nanoparticles realized (5-FU/MSNs, HA-FA/5FU-MSNs, CMCH-DA/5FU-MSNs, SI/MSNs, HA-FA/SI-MSNs, and CMCH-DA/SI-MSNs). The residual content of 5-FU and silymarin was determined with a UV-Vis spectrophotometer (ThermoFisher Scientific, Waltham, MA, USA) at 265 nm and 286 nm, respectively. The loading efficiency and loading capacity of both drugs were calculated with Equation (1):

(1) loading efficiency = initial amount of drug − amount of drug in supernatant × 100initial amount of drug

The amount of 5-FU and Si used for loading was 30 mg.

### 2.7. In Vitro Release Profile

The release of 5-FU and silymarin da was determined in three phosphate-buffered saline (PBS) media with pH values of 5.5, 6.8, and 7.4 to simulate normal and tumor environments using dialysis membranes with cut-offs of 12,000/14,000 kDA. A total of 5% DMSO was added to the release medium to facilitate drug solubilization. Briefly, a certain amount of coated and uncoated nanoparticles (15 mg) was weighed for insertion into each dialysis bag. A total of 2 mL of each medium was poured into each dialysis bag and each was placed in a 50 mL container of the same medium at the created pH [22]. These dispersions of MSNs were shaken at 37 °C under magnetic stirring in a sealed light condition. At predetermined time intervals (1, 2, 4, 6, 24, 48, and 72 h), the entire suspension was withdrawn and replaced with a fresh medium to continue the release profile. The amounts of 5-FU and silymarin were determined using a UV-Vis spectrophotometer [22].

### 2.8. Antitumor Activity

#### 2.8.1. Cell Culture

The human breast cancer epithelial cell line MCF-7 (estrogen receptor (ER α-positive) was cultured in DMEM-F12 containing 10% fetal bovine serum (FBS), 1% penicillin/streptomycin, and 1% L-Glutamine. T98G is a human glioblastoma multiforme cell line and was maintained in MEM 1× (GIBCO) supplemented with 10% FBS, 1% penicillin/streptomycin, 1% L-Glutamine, 1% Sodium Pyruvate, and 1% non-essential amino acids. Both cells were acquired from ATCC and were grown in an incubator a temperature of 37 °C in the presence of 5% CO_2_.

#### 2.8.2. MTT Assay

MCF-7 and T98G cells were plated in a 96-well plate at a confluence of 5000 cells/well and grown in complete media. Before starting the proliferation assay, cells were starved in an FBS-free medium for 24 h. At the end of starvation, cells were treated with free drugs (Si, 5-FU), free polymeric matrices (HA-FA, CMCH-DA), empty MSNs, and all typologies of nanoparticles (5-FU/MSN, HA-FA/5-FU MSN, CMCH-DA/5-FU MSN, Si/MSN, HA-FA/Si MSN, and CMCH-DA/Si MSN) for 24, 48, and 72 h. After this, the treatment was removed and 100 μL of MTT was added and incubated for 3 h. A total of 100 μL of DMSO (Dimethyl-sulfoxide) was used to replace the MTT reagent and was left to incubate for 30 min. The reduction from MTT to formazan (resulting in a colored solution) by viable cells was quantified by measuring the absorbance at 500–600 nm using a 96-well plate spectrophotometer (Muliskan SkyHigh, ThermoFisher Scientific, Waltham, MA, USA).

### 2.9. Statistical Analysis

Data were analyzed by an unpaired *t*-test and by a one-way analysis of variance (one-way ANOVA) followed by Tukey’s test.

## 3. Results

### 3.1. Preparation and Characterization of Coated MSNs

**Figure 1:**
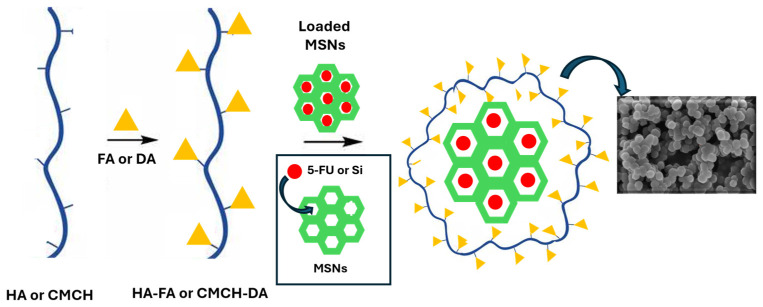
Schematic illustration of coated and loaded MSNs.

MSNs capable of encapsulation of 5-FU and silymarin were synthesized by a sol–gel synthetic strategy using CTAB, a cationic surfactant template, and an aqueous solution consisting of TEOS, a silica source (Figure 1). Coated MSNs were also obtained using two different polymeric derivatives, HA-FA and CMCH-DA, able to provide targeted delivery of the selected antitumor agents into tumor cells. In fact, the surface polymer coated on nanocarriers was sensitive to environmental pH, as well as to overexpressed substances in the tumor site. The synthesized nanoparticles were characterized to evaluate their morphology and structural arrangement by different analytical techniques like FT-IR, DLS, XRD, and SEM. The results of the characterization of all synthesized MSNs are described below.

### 3.2. FT-IR Spectra

**Figure 2:**
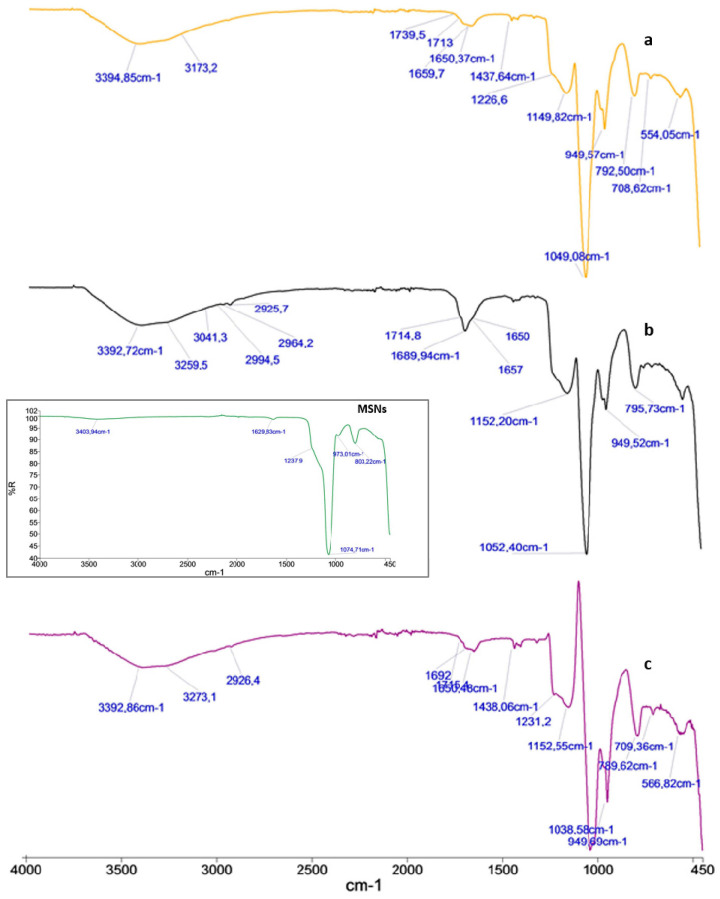
FT-IR Spectra of 5-FU/MSNs (**a**), HA-FA/5-FU-MSNs (**b**), and CMCH-DA/5-FU-MSNs (**c**).

FT-IR spectra of the prepared particles were measured over the range 4000 to 450 cm^−1^. The particles formation was confirmed by FT-IR (Figure 2). In particular, the spectra of MSNs (Figure in black box), loaded 5-FU (Figure 2a), HA-FA/5FU-MSNs (Figure 2b), and that of the CMCH-DA/5FU-MSNs (Figure 2c) were compared. The Si-O bond stretching of surface MSNs Si-OH groups was shown at 973 cm^−1^. Additionally, the very broad hydroxyl stretching band for the silanol Si-O-H hydroxyl group was evident at 3403 cm^−1^. In addition, the internal Si-O-Si stretching vibration of the SiO_4_ asymmetric band appeared at 1073 cm^−1^, while the symmetric one was at 803 cm^−1^ (see the spectrum in the black box). The spectrum of the 5-FU loaded MSNs (Figure 2a) showed a broad band between the 3000 and 3500 cm^−1^ and two bands in the range of 1620–1670 cm^−1^ that were attributed to—NH and carbonyls stretching (C=O) vibrations of 5-FU. The peak at 1226 cm^−1^ was due to the C-F stretching band of 5-FU. Concerning the HA-FA/5-FU-MSNs spectrum (Figure 2b) it showed a broad band between 1600 and 1750 cm^−1^ corresponding also to the stretching of the carbonyl group of the ester and a sharper and broader band at 3200–3500 cm^−1^, corresponding to the –OH groups stretching vibrations of FA and HA, confirming the successful derivatization. The FT-IR spectrum of the CMCH-DA/5-FU-MSNs exhibited a similar trend (Figure 2c).

### 3.3. Morphological Analysis of MSNs

**Figure 3:**
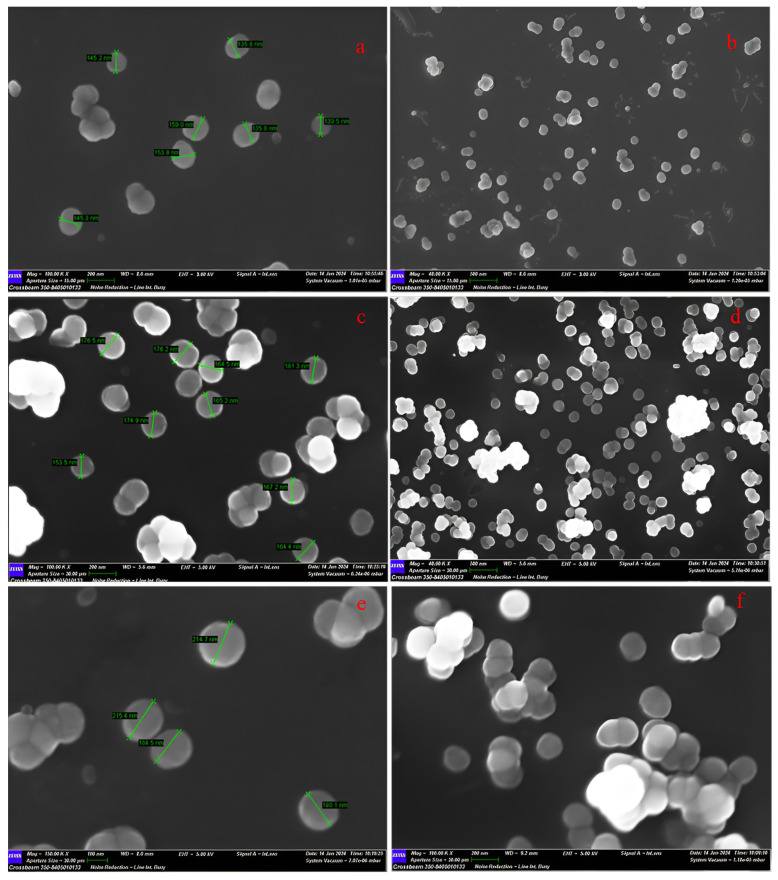
SEM image of MSNs (**a**,**b**), 5-FU/MSNs (**c**,**d**), and HA-FA/5-FU-MSNs (**e**,**f**).

Based on the SEM image of all the prepared particles, the samples were uniform, with good dispersion and a spherical shape as shown in Figure 3.

In particular, for empty MSNs, the SEM image showed the size of particles to be about 125–160 nm (Figure 3a,b). Instead, the SEM image of 5-FU MSNs revealed an increase in sizes of about 153–182 nm (Figure 3c,d). Finally, the insertion of polymeric coating caused an additional dimension rise, as revealed in Figure 3e,f in the case of hyaluronic acid esterified with folic acid (HA-FA) (180–216 nm). The same behavior was observed both for MSNs coated with carboxymethyl chitosan esterified with dopamine (CMCH-DA) and for particles containing silymarin.

The particle size distribution of the MSNs was also determined by DLS to provide the hydrodynamic diameter of the empty MSNs and those loaded with the drug and/or coated with HA-FA and CMCH-DA (Table 2). The coating layer with polymeric matrices increased the overall particle size compared to MSNs without the coating layer. The results are in good agreement with those obtained from SEM images of the MSNs (Figure 3). However, the differences in nanoparticle size between the DLS and SEM measurements can be attributed to the hydrodynamic radius and water-inflated nanoparticles for the DLS measurement, whereas SEM represents an estimate of the nanoparticle diameter in a dried state.

**Figure 4:**
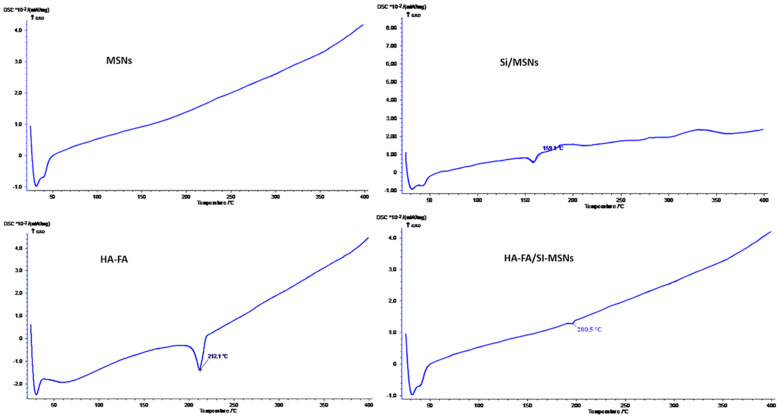
DSC curves of MSNs, SIMSNs, HA-FA, and HA-FA/MSNs.

The DSC curve of silymarin-loaded MSNs (Si/MSNs) showed, compared to empty MSNs, a small and broad endothermic peak at 159.1 °C related to the melting of the bulk active substance, suggesting that part of the silymarin is confined in the pore subjected to crystallization. Regarding the coated MSNs, only a smaller endothermic peak (like the HA-FA one) was observed at 200.5 °C due to the melting of the functional polymer (Figure 4).

### 3.4. XRD Analysis

**Figure 5:**
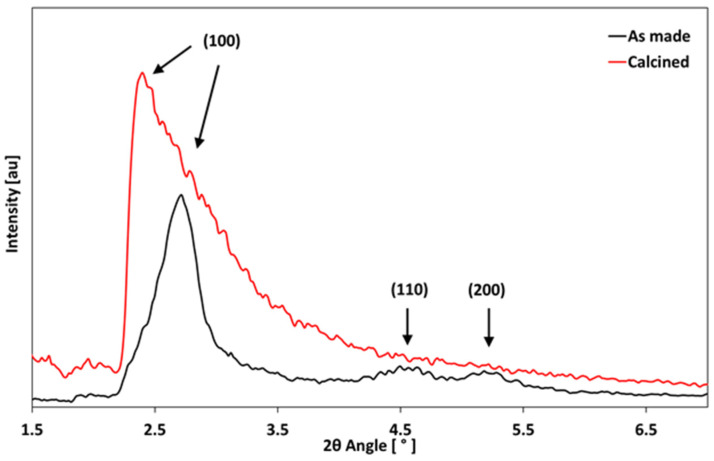
XRD pattern of MSNs calcined and MSNs after the removal of the template.

The XRD analyses confirmed the formation of a mesoporous structure. The pore order was preserved after the removal of organic components by calcination. The powder underwent mild pore shrinkage; in fact, the (100) peak shifted from 2.40° in the as-made sample to 2.72° in the calcined sample, as highlighted by the arrows in Figure 5. This is due to the slight collapse of the silica walls. The (110) and (200) peaks were detected in the as-made powder at c.a. 4.60° and 5.26, respectively.

### 3.5. Drug Entrapping Profile

Drug loading into MSNs is generally influenced by the pore size and chemical nature of the drug and coating used. The loading efficiency of 5-fluorouracil and silymarin was calculated with a UV-visible spectrophotometer (Table 3). The results showed that the loading efficiency percentage of 5-fluorouracil and silymarin was 73.3% and 66.6% for MSNs, respectively. In the presence of polymers, the loading efficiency changed. In particular, it increased (89%) using HA-FA as a functional polymer and 5-FU as a drug and decreased in the case of CMCH-DA as a polymer (71,6%). In contrast, when using silymarin as a drug, the effectiveness decreased with the hyaluronic acid-based coating (56.6%) and increased with the carboxymethyl chitosan-based one (72%). This behavior could be attributed to the different affinity between drugs and the type of polymeric matrix used to coat the silica nanoparticles.

### 3.6. In Vitro Drug Release

**Figure 6:**
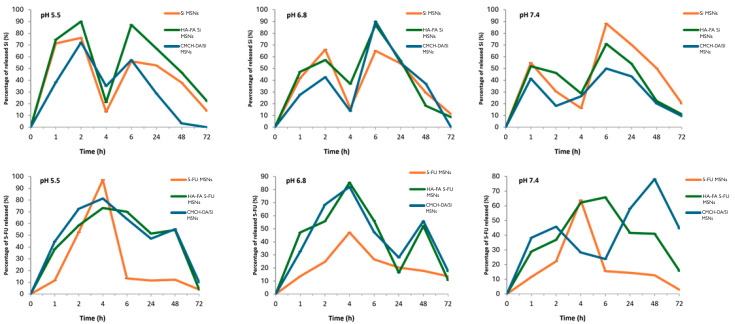
5-FU and Si release profiles evaluated within 72 h.

The release profiles of 5-FU and silymarin from 5-FU/MSNs, HA-FA/5-FU-MSNs, CMCH-DA/5-FU-MSNs, SI/MSNs, HA-FA/SI-MSNs, and CMCH -DA/SI-MSN, are shown in Figure 6 and are correlated to three different pH values (5.5, 6.8, and 7.4). The release of both 5-FU and silymarin from the materials was found to be pH-sensitive. A similar trend was observed for all types of prepared materials and a greater percentage of release at slightly acidic pHs, typical of tumor environments.

### 3.7. Antitumor Activity

**Figure 7:**
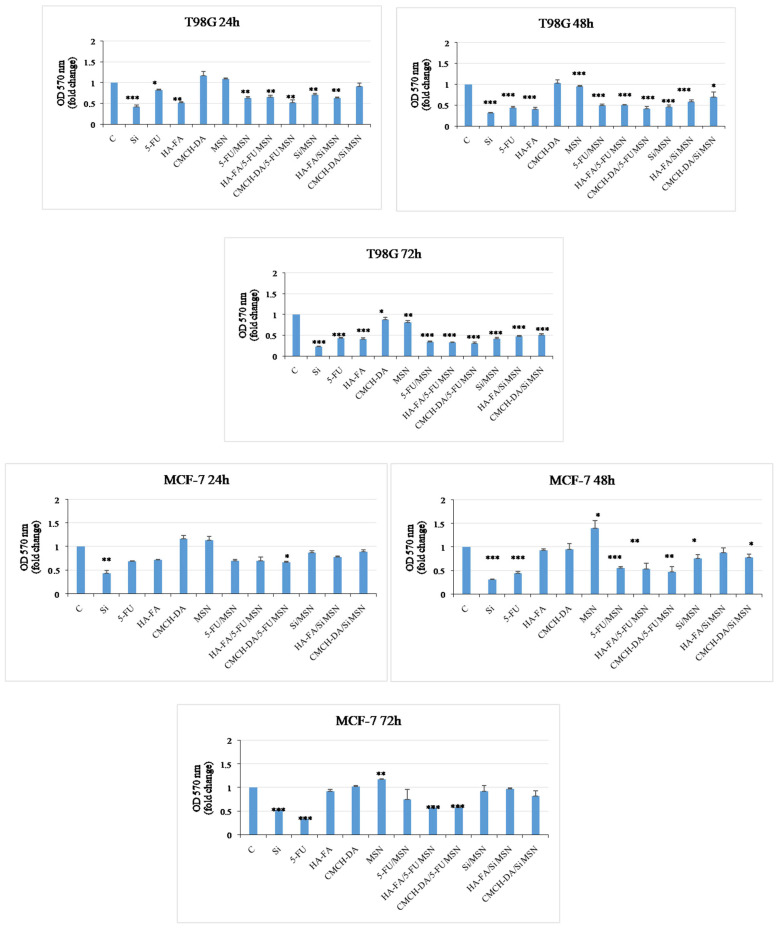

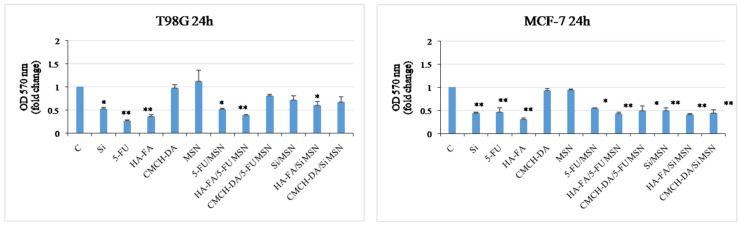
Effects of prepared materials on breast cancer cell and on glioblastoma cell line growth. MTT growth assays in MCF-7 and T98G cells treated with all obtained particles for 24, 48, and 72 h. Cell proliferation is expressed as the fold change ± S.D. relative to control (C) cells and is representative of three different experiments each performed in triplicate. (* *p* < 0.05, ** *p* < 0.01, and *** *p* < 0.001).

Glioblastoma multiforme is the most aggressive cancer type in adults. Despite the current treatment methods, such as chemical and surgical operations, the prognosis is still poor. The average survival rate for patients is around 15 months, with a high probability of disease recurrence. Therefore, the development of new therapeutic strategies is important [23,24]. Breast cancer is the most commonly diagnosed malignancy and the leading cause of cancer death in women worldwide. Depending on the receptor status, breast cancer is treated with surgery and adjuvant chemotherapy, which includes SERMS, SERDs, and aromatase inhibitors. Nevertheless, breast cancer is known to develop resistance to drug treatment over time [25,26,27,28,29]. To overcome resistance mechanisms, new therapeutics must be explored to improve efficacy. We evaluated the impact of free drugs (Si, 5-FU), free polymeric matrices (HA-FA, CMCH-DA), empty MSNs, and all typologies of nanoparticles (5-FU/MSN, HA-FA/5-FU MSN, CMCH-DA/5-FU MSN, Si/MSN, HA-FA/Si MSN, and CMCH-DA/Si MSN) on breast cancer cell viability using ER-α positive MCF-7 on glioblastoma cell line T98G. For the treatments, we used a3 µg/µL concentration of each substance. MTT assays were performed to test cell viability. A significant decrease in cell viability was observed in T98G cells after treatment with Si, 5-FU, HA-FA, 5-FU/MSN, and HA-FA/5-FUMSN. However, we saw less reduction than in untreated cells in T98G cells treated with CMCH-DA/5-FU MSN, Si/MSN, and HA-FA/Si MSN8 after 24 h. In addition, we also observed a significant decrease in proliferation in glioblastoma cells treated with HA-FA, whereas cells treated with compounds CMCH-DA and MSN showed a trend similar to that of control cells (Figure 7). In breast cancer cells, we observed a significant reduction in samples treated with compounds Si, 5-FU, HA-FA, 5-FU/MSN, HA-FA/5-FU MSN, CMCH-DA/5-FU MSN, Si/MSN, HA-FA/Si MSN, and CMCH-DA/Si MSN. In contrast, cells treated with CMCH-DA and empty MSNs showed levels comparable to control cells after 24 h (Figure 7). MTT assays were performed to test cell viability. The results we obtained showed a significant reduction in the proliferation of glioblastoma cells treated with Si, 5-FU, HA-FA, 5-FU/MSN, and HA-FA/5-FUMSN in a time-dependent manner. On the other hand, cells treated with CMCH-DA and empty MSNs showed a level of growth similar to that of control cells. In breast cancer cells, we observed a significant time-dependent reduction in cell growth in MCF-7 cells treated with compounds Si, 5-FU, 5-FU/MSN, HA-FA/5-FU MSN, and CMCH-DA/5-FU MSN, but not with Si/MSN, HA-FA/Si MSN, and CMCH-DA/Si MSN compared to control. In contrast, we observed a time-dependent increased growth in cells treated with HA-FA, CMCH-DA, and empty MSNs.

## 4. Conclusions

The aim of this work was the design, preparation, and study of coated MSNs potentially useful as delivery systems for 5_FU and Si. MSNs, obtained through the sol–gel strategy, were coated using two different polymeric matrices, HA-FA and CMCH-DA, with the aim to enhance the aqueous solubility of selected drugs and improve loading capacity, therapeutic efficacy, and sustained release. The results showed a good loading capacity between 56 and 89%, good biocompatibility, and a pH-dependent in vitro release profile. These results confirmed selective targeting and successful delivery of 5-FU and Si by the designed MSNs. In particular, these findings suggest that all materials (HA-FA/5-FU-MSNs, HA-FA/Si-MSNs, CMCH-DA/5-FU-MSNs, and CMCH-DA/Si-MSNs) could be promising candidates for targeted anticancer delivery, mainly to glioblastoma and beyond that, breast cancer, with reduced cell viability action 24 h after treatment. Although the potential of MSNs as anticancer drug delivery agents is high, the understanding of its behavior in the human body is still limited. It is important to be able to delve deeper into the biosafety problem related to the evaluation of renal clearance, toxicity within the human body, and administration efficacy. Furthermore, it is necessary to delve deeper into large-scale aspects, such as the development of industrial synthesis routes that produce large quantities of high-quality MSNs with batch-to-batch reproducibility.

## Figures and Tables

**Table 1:** Amounts of substrates used.

Sample	MSN	5-Fluoruracil	Sylimarin	HA-FA	CMCH-DA
5-FU/MSNs	30 mg	30 mg	-	-	-
HA-FA/5-FU-MSNs	30 mg	30 mg	-	2%	-
CMCH-DA/5-FU-MSNs	30 mg	30 mg	-	-	2%
SI/MSNs	30 mg	-	30 mg	-	-
HA-FA/SI-MSNs	30 mg	-	30 mg	2%	-
CMCH-DA/SI-MSNs	30 mg	-	30 mg	-	2%

**Table 2:** MSNs DLS analysis.

Formulation	Size (nm)	Polydispersion Index (PDI)
MSNs	185.2 ± 1.3	0.183
5-FU/MSNs	200.8 ± 0.3	0.225
HA-FA/5-FU-MSNs	227.2 ± 1.2	0.251
CMCH-DA/5-FU-MSNs	238 ± 2.1	0.214
SI/MSNs	202.5 ± 0.7	0.246
HA-FA/SI-MSNs	232.2 ± 0.5	0.239
CMCH-DA/SI-MSNs	241.1 ± 1.4	0.278

**Table 3:** MSNs loading efficiency.

Formulation	Loading Efficiency (%)
5-FU/MSNs	73.3%
HA-FA/5-FU-MSNs	89%
CMCH-DA/5-FU-MSNs	71.6%
SI/MSNs	66.6%
HA-FA/SI-MSNs	56.6%
CMCH-DA/SI-MSNs	72%

## Data Availability

Data are contained within the article.
